# Prevalence of hospital malnutrition among cardiac patients: results from six nutrition screening tools

**DOI:** 10.1186/2193-1801-3-412

**Published:** 2014-08-07

**Authors:** Anidu K Pathirana, Niroshan Lokunarangoda, Ishara Ranathunga, Wijeyasingam Samuel Santharaj, Ruwan Ekanayake, Ranil Jayawardena

**Affiliations:** Institute of Cardiology, National Hospital of Sri Lanka, Colombo, Sri Lanka; Department of Medicine, Faculty of Medicine and Allied Sciences, Rajarata University of Sri Lanka, Mihintale, Sri Lanka; Institute of Health and Biomedical Innovation, Queensland University of Technology, Brisbane, QLD Australia; Diabetes Research Unit, Department of Clinical Medicine, Faculty of Medicine, University of Colombo, Colombo, Sri Lanka

**Keywords:** Malnutrition, Cardiac patients, Developing country, Nutrition screening tools, Sri Lanka

## Abstract

Malnutrition is highly prevalent among hospitalized patients, ranging from 30% to 50% depending on the patient population and the criteria used for diagnosis. Identifying early those who are malnourished and at risk of malnutrition and intervening at an early stage will improve patients overall prognosis and will reduce the costs to the state. Even though cardiac patients are at risk of malnutrition, data on the prevalence of malnutrition among cardiology inpatients is limited. The aim of the study was to assess malnutrition status of the newly admitted patients in a specialist cardiology institution in Sri Lanka by internationally recommended nutrition screening and assessment tools. During study period, 322 (61.22%) males and 204 (38.78%) females were recruited. Malnutrition status assessed by each screening tool had a wide variation. According to Mini Nutritional Assessment tool 69.6% were having possible malnutrition. Malnutrition Screening Tool 47.9% to be at risk of malnutrition. Subjective Global Assessments categorized only 4.2% as malnourished on the other hand Malnutrition Universal Screening Tool categorized 20.4% and 19.6% subjects as at medium risk and high risk of malnutrition respectively. Nutritional Risk Screening detected 6.3%, 25.1% and 24.9% patients to be mildly, moderately and severely malnourished respectively. Short Nutrition Assessment Questionnaire categorized 5.0% and 17.7% patients to be moderately malnourished and severely malnourished correspondingly. In conclusion, Although malnutrition was prevalent among this population, a wide variation in the nutritional status when assessed by widely used internationally recognized tools was observed.

**Electronic supplementary material:**

The online version of this article (doi:10.1186/2193-1801-3-412) contains supplementary material, which is available to authorized users.

## Introduction

Malnutrition is any imbalance in nutrition; from over-nutrition to under-nutrition. Over-nutrition which was more prevalent in developed countries is now becoming a major health problem in less developed countries as well (Subramanian and Smith 2006). Under-nutrition can occur as a consequence of deficiency in dietary intake, poor absorption, increased requirements or from excessive nutrient losses associated with the disease state or from a combination of above factors (Soeters et al. 2008). Negative consequences of malnutrition can be varied: longer length of hospital stay, increased morbidity and mortality (Reilly et al. 1988) affecting patients and increased health expenditure for the state (Neumayer et al. 2001). Despite current advancements in understanding of the value of proper nutritional care, the malnutrition is yet highly prevalent among hospitalized patients, ranging from 30% to 50% depending on the patient population and the criteria used for diagnosis (McWhirter and Pennington 1994) and is often unrecognized and underestimated by health care workers (Stratton et al. 2006). Identifying early those who are malnourished and at risk of malnutrition and intervening at an early stage will improve patients overall prognosis and will reduce the costs to the state (Neumayer et al. 2001). Therefore many validated tools for nutrition risk screening and nutrition assessment exist for the accurate identification, referral and treatment of patients who are malnourished or at risk of malnutrition. Nutrition screening is a process of identifying characteristics known to be associated with malnutrition risk while nutrition assessment is a diagnostic tool to determine if a patient is currently malnourished (Identifying patients at risk: ADA's definitions for nutrition screening and nutrition assessment Council on Practice COP Quality Management Committee 1994).

Many nutrition screening and assessment tools exist to identify risk of, and diagnose, malnutrition. The Malnutrition Screening Tool (MST) is a simple instrument to identify patients at high risk of malnutrition (Ferguson et al. 1999). Related to the MST, the Malnutrition Universal Screening Tool (MUST) was developed to detect both under-nutrition and obesity in adults (Stratton et al. 2006). The Nutritional Risk Screening (NRS) is the preferred screening tool for hospitalized patients (Kondrup et al. 2003). Mini Nutritional Assessment – the Short Form (MNA-SF) was developed to assess nutritional risk in elders (Guigoz 2006). The four item Short Nutrition Assessment Questionnaire (SNAQ) was developed to diagnose malnutrition in hospitalized patients and does not require calculation of BMI (Kruizenga et al. 2005a). Subjective Global Assessment (SGA) is one of the most commonly used nutrition assessment tools and reliably detects patients with established malnutrition (Detsky et al. 1987). Ideally, nutritional assessment should be practical, easy to perform, non-invasive, well tolerated, inexpensive, requiring no use of devices or supplementary examinations, applicable at the bedside, show appropriate sensitivity and specificity and yield immediate result.

Prevalence of malnutrition among children, general medical and surgical inpatients has been widely reported. However, data on the prevalence of malnutrition among cardiology inpatients is limited (Yamauti et al. 2006). Lomivorotov et al. reported that significant prognostic values of different nutritional screening tools in patients undergoing cardiopulmonary bypass (Lomivorotov et al. 2013). Malnutrition is common among cardiology wards due to heart failure, anorexia, pre-investigate ‘nil by mouth’ and due to cardiac cachexia (Webb et al. 1986). Although it is highly recommended to screen for malnutrition upon cardiac admission, low percentage of screening is reported even in the developed world (Joyce et al. 2011). In some countries, including United Kingdom, United States, the Netherlands and some parts of Denmark, nutrition screening on patient admission is mandatory (Elia et al. 2005).

Currently, ischemic heart disease (IHD) is the leading cause of mortality in hospitals in Sri Lanka accounting for 22.7 deaths per 100000 population while it has led to 330 admissions per 100,000 of all hospital admissions (Ministry of Healthcare and Nutrition Sri Lanka 2009). A proper malnutrition assessment is not being carried out in this subgroup of patients. Aim of this study was to assess the nutritional status of patients with suspected or proven cardiological diagnosis and to establish the prevalence of malnutrition in this patient population, using MST, MUST, SNAQ, MNA-SF, NRS and SGA screening tools in the national level tertiary care institute in Sri Lanka.

## Methods

Consecutive patients admitted to the cardiology unit of a tertiary care hospital were enrolled from March 2012 to July 2012. Planned admissions for investigations or interventions, patients whose medical condition prevented them from having their anthropometric measurements taken or patients who were unable to complete the questionnaire were excluded. Moreover, patients with acute oleander poisoning, pregnant and lactating women were not involved in the study. This questionnaire included socio-demographic data, medical history of current disease status, subjective assessment of the nutritional status using patients’ history and examination and objective assessment through anthropometric measurements. All data collection was done by a specially-trained medical officer with close supervision of clinical nutrition and cardiology experts. All patients who provided informed written consent were screened for malnutrition using an interviewer administered questionnaire.

Weight (to the nearest 100 g) was measured using a electronic flat scale (seca 815, seca GmbH. Co. kg, Germany) and height (to the nearest 1 mm) was measured using a standard stadiometer (Stadiometer for mobile height measurement - seca 217, seca GmbH. Co. kg, Germany) by a trained medical officer. Body mass index (BMI) was calculated by dividing weight (kilograms) by the square of height (meter).

Nutritional status was assessed by MST, MUST, SNAQ, MNA-SF, NRS and SGA questionnaires. MST is a simple, three-question tool assessing recent unintentional weight and appetite loss. MUST assess body mass index, unplanned weight loss in past 3–6 months and the presence or absence of acute illness or lack of nutritional intake >5 days. NRS 2002 includes reduced BMI, recent weight loss, recent decreased dietary intake and severity of the illness. MNA-SF utilizes information on decreased food intake, recent weight loss, degree of mobility, recent psychological stress, acute illness, neuropsychological problems and BMI. SNAQ assessed recent weight loss, recent decreased appetite, and recent intake of supplemental drinks or tube feeding. SGA was used to assess nutrition using data on weight change, dietary intake change, gastrointestinal symptoms (dysphagia, nausea, vomiting, diarrhoea, anorexia), and changes in functional capacity (normal, suboptimal, bedridden) in relation to malnutrition as well as assessment of fat and muscle stores and the presence of oedema and ascites by a trained medical officer. The diagnosis made by the clinicians was utilized to calculate diagnosis related stress level measured in SGA.

Data entry and statistical analysis was performed using the SPSS Version 16.0 statistical package. Categorical variables were expressed as number and percentage (%) and continuous variables were expressed as mean and standard deviation. Chi square test and *t*-test were used to compare male and female population. P value < 0.05 was considered as statistically significant. Patients were classified in to different nutritional groups according to each different tool.

The present study was conducted according to the guidelines laid down in the Declaration of Helsinki and was approved by the Review Committee, National Hospital of Sri Lanka, Sri Lanka.

## Results

Five hundred twenty six patients were included, of whom 322 (61.22%) were males and 204 (38.78%) were females. The majority were Sinhalese (n = 438, 83.3%) while Sri Lankan Tamils (n = 43, 8.2%), Indian Tamils (n = 4, 0.8%), Muslims (n = 34, 6.5%) and other ethnic groups (n = 7, 1.3%) made the rest of the population. A large proportion (n = 172, 32.7%) had studied up to grade 11 and only 55 (10.5%) had not had a formal education. The population consisted of a variety of patients with the majority presenting with an acute coronary syndrome (n = 275, 52.3%). Arrhythmias was the second commonest disease category and included 67 (12.7%) patients., Fifty nine patients presented with heart failure and cardiomyopathies while another Fifty nine patients (11.2%) were categorized under miscellaneous (Table 1). The mean age was 58.5 ± 12.0 years. There was a significant difference of age between males (M = 57.6, SD = 12.5) and females (M = 59.9 ± 11.2, p = 0.04). Ages ranging from 22 years to 85 years were included in the study and the majority was in the age group of 50–60 years. The mean body mass index (BMI) was 23.61 kgm^-2^ (±4.15) while the females had a higher BMI than males (males 23.33 ± 3.67, females 24.03 ± 4.80; p = 0.07).

**Table 1:** **Socio-demographic and clinical characteristics of patients in the population**

Patient characteristic	Population (n = 526)		Males (n = 322)		Females (n = 204)	
	Number	%	Number	%	Number	%
Ethnicity						
Sinhala	438	83.3	266	82.6	172	84.3
Muslim	43	8.2	24	7.5	19	9.3
Indian Tamil	4	0.8	4	1.2	0	0
Sri Lankan Tamil	34	6.5	22	6.8	12	5.9
Other	7	1.3	6	1.9	1	0.5
Education level						
Not Educated	55	10.5	25	7.8	30	14.7
Up to Grade 5	175	33.3	96	29.8	79	38.7
Up to Grade 11	172	32.7	113	35.1	59	28.9
Up to Grade 13	101	19.2	73	22.7	28	13.7
Tertiary	23	4.4	15	4.7	8	3.9
Age Categories						
20-30	8	1.5	7	2.2	1	0.5
30-40	40	7.6	28	8.7	12	5.9
40-50	82	15.6	54	16.8	28	13.7
50-60	158	30.0	99	30.7	59	28.9
60-70	153	29.1	85	26.4	68	33.3
>70	85	16.2	49	15.2	36	17.7
Diagnosis Categories						
ACS	275	52.3	188	58.4	87	42.6
Acute non-cardiac infections	17	3.2	10	3.1	7	3.4
Anaemia	6	1.1	2	0.6	4	2.0
Arrhythmias	67	12.7	36	11.2	31	15.2
HF/Cardiomyopathy	59	11.2	33	10.2	26	12.7
Infective endocarditis	5	1.0	4	1.2	1	0.5
Pericardial disease	7	1.3	3	0.9	4	2.0
PHT	10	1.9	1	0.3	9	4.4
Respiratory disease	5	1.0	3	0.9	2	1.0
Valvular heart disease	16	3.0	7	2.2	9	4.4
Miscellaneous	59	11.2	35	10.9	24	11.8
Age	58.5 ± 12.0		59.9 ± 11.2		57.6 ± 12.5	
BMI	23.33 ± 3.67		24.03 ± 4.80		23.60 ± 4.15	

Malnutrition status assessed by each screening tool had a wide variation. Number and percentage of patients falling under each parameter assessed in each tool is shown in Table 2. MNA, MST, SGA and NRS tools could be applied to the entire population since a response could be obtained for every parameter assessed while MUST, SNAQ were applicable only to a proportion of the population as a response could not be attained for all the parameters used in the above tools. MUST screened only 113 (21.5%) patients as the rest of the population (n = 413, 87.5%) were not able to recall unplanned weight loss in the past 3–6 months. As 407 (77.4%) patients were incapable of recalling their lost weight within last 1–6 months, SNAQ were applicable to only a subset of patients (n = 119; 22.6%).

**Table 2:** **Prevalence of malnutrition according to malnutrition screening and assessment tools**

	Score		Number	%
MNA (n = 526)	12-14	Normal	160	30.4
	8-11	At risk of malnutrition	336	63.9
	0-7	Malnourished	30	5.7
MST (n = 526)	<2	No risk of malnutrition	276	52.1
	> = 2	Risk of malnutrition	250	47.9
SGA (n = 526)	<17	Well nourished	503	95.8
	17 - 22	Moderately malnourished	22	4.0
	>22	Severely malnourished	1	0.2
NRS (n = 526)	0	Normal	230	43.7
	1	Mild	33	6.3
	2	Moderate	132	25.1
	3	Severe	131	24.9
MUST (n = 113)	0	Low Risk	68	60.2
	1	Medium Risk	23	20.4
	> = 2	High Risk	22	19.6
SNAQ (n = 119)	<2	Well nourished	92	77.3
	2	Moderately malnourished	6	5.0
	> = 3	Severely malnourished	21	17.7

According to MNA, which divided the sample population in to three groups, 160 (30.4%) patients were identified as having normal nutritional status, 336 (63.9%) as at risk of malnutrition and 30 (5.7%) as malnourished. MST detected 276 (52.1%) subjects to be normal and 250 (47.9%) to be at risk of malnutrition. SGA categorized 503 (95.8%) subjects as well-nourished and 23 (4.2%) as malnourished. Although the cumulative value of SGA was low, the sub-components of SGA relived nearly every second patient had lost weight unintentionally (Additional file 1). NRS divided patients in to malnutrition categories of normal (230; 43.7%), mild (33; 6.3%), moderate (132; 25.1%) and severe (131; 24.9%) respectively. However, most of NRS scores were derived from food intake data rather than weight lose percentages. MUST categorized 68 (60.2%), 23 (20.4%) and 22 (19.6%) subjects as at low risk, at medium risk, and at high risk of malnutrition levels. SNAQ categorized 92 (77.3%), 6 (5.0%). 21(17.7%) patients to be well nourished, moderately malnourished and severely malnourished correspondingly.

Distribution of the patients in the sub component of the each nutrition screening and assessment tool is presented in Additional file 1 as online supplementary file.

## Discussion

To the best of our knowledge, this is the first study done to assess the prevalence of malnutrition in general cardiac patients by means of widely used internationally recommended six malnutrition screening tools. Furthermore this is also the first study done on Sri Lankan hospital patients to assess the prevalence of malnutrition.

Prevalence of malnutrition among different group of population such as paediatric (Sermet-Gaudelus et al. 2000; Secker and Jeejeebhoy 2007), geriatric (Sacks et al. 2000; Stratton et al. 2004; Rubenstein et al. 2001) and adult inpatients (Stratton et al. 2004) has been broadly studied. Moreover, malnutrition among different specialties of internal medical (Stratton et al. 2004), surgical (Detsky et al. 1987; Stratton et al. 2004), oncology (Bauer et al. 2002) and acute medicine also has been evaluated. Cardiac inpatients is another risk group which is highly prone for malnutrition due to most apparent reasons such as heart failure, anorexia, pre-investigate ‘nil by mouth’, due to cardiac cachexia (Webb et al. 1986). Nevertheless, publications on the prevalence of malnutrition among adult cardiology inpatients are limited (Yamauti et al. 2006). Pirlich et al. reported over 20% of malnutrition among cardiology patients in Germany (Pirlich et al. 2006).

Present study recruited consecutive patients admitted to the cardiology unit of a tertiary care hospital, ensuing a large subject population with accurate representation of the study population. In this study, malnutrition status assessed by each tool had a wide variation ranging from 4.4% to 69.6% detected by SGA and MNA-SF respectively. We have reported responses for each component of the different tools (Additional file 1). A wide variety of risk factors that is considered in each malnutrition risk assessment tool, ranging from objective measurements to subjective assessment might be accountable to different categorizations obtained from each tool (Green and Watson 2006). Moreover, even when different malnutrition screening tools detects equivalent percentage to be malnourished, the subjects identified at risk may differ (Stratton et al. 2004). Even though literature illustrates the differences in classification of nutritional status by different screening tools, Stratton et al. illustrates that MUST has excellent agreement with NRS and SGA, fair amount of agreement with and MST and MNA-SF (Kyle et al. 2006). When comparing MUST and SNAQ, MUST had been found to have higher accuracy to detect malnutrition than SNAQ measured by low fat free mass index in a study carried out on patients undergoing cardiac surgery at 59% and 19% prevalence respectively (van Venrooij et al. 2011). When comparing MST with SGA in a study done on oncology patients receiving chemotherapy, MST has acceptable relative validity, inter-rater reliability, sensitivity, and specificity, and hence proved to be an acceptable nutrition screening tool to detect malnutrition (Isenring et al. 2006). In a study carried out in hospitalized patients in Brazil to identify the most appropriate nutritional screening tool for predicting unfavourable clinical outcomes, NRS, MUST, and MNA-SF detected malnutrition risk in 27.9%, 39.6%, and 73.2% of patients, respectively. NRS and MNA-SF found to have similar sensitivity to predict clinical outcomes, though NRS seems to provide the best yield (Raslan et al. 2010). In an evaluation of three nutritional screening tools in a Portuguese oncology centre, MUST identified the highest proportion of nutritionally-at-risk patients (43.8%), followed by 28.5% using NRS-2002 and 17.7% using MST (Amaral et al. 2008). Similar to above, present study also identified a wide variation in the malnutrition status when measured from each different tool, even though, the similarities do exist (MST 47.9%, MUST 40%, NRS 44.3%).

According to local (Katulanda et al. 2010) and regional evidence (WHO Expert Consultation 2004) (Misra et al. 2009) the lower BMI values are recommended for South Asians. Since number of malnutrition assessment and screening (MUST, NRS, MNA, SNAQ, MST) tools utilizes BMI cut offs and weight loss values that is suitable for Caucasians, for which the tools are originally developed the above assessment and screening tools may categorize South Asians to be falsely malnourished.

SGA was originally developed and validated to assess malnutrition risk in gastrointestinal surgical patients (Detsky et al. 1987). Moreover, it is proved to be useful to assess malnutrition in different populations and to predict prognosis in numerous clinical situations (Barbosa-Silva and Barros 2006). Nevertheless, Agreement between subjective global assessment and some other malnutrition screening methods is not always acceptable (Barbosa-Silva and Barros 2006). Prevalence of malnutrition varies according to the population studied, such as 19.2% of stroke patients (Martineau et al. 2005), 76% of oncology patients (Bauer et al. 2002), 80% of liver transplant candidates (Hasse et al. 1993), 69.8% of geriatric residents (Sacks et al. 2000), 51% of paediatric population (Secker and Jeejeebhoy 2007), 47.6% of medical patients (Baccaro et al. 2007) and 51.9% of cardiac patients (Yamauti et al. 2006). Present study which was done on 526 cardiac patients identified 4.4% (n = 24) to be malnourished mainly due to low/nil responses to some parameters (Supplementary documents, Additional file 1). Even though MNA-SF has been validated to be used in the geriatric population, its use on general medical patients is still need to be explored although we had a portion of (n = 238) of over 60 years old patients (Rubenstein et al. 2001). MNA-SF identified 366 (69.6%) patients to be at risk of possible malnutrition and screened the whole population as it did not require details on previous weight measurements. MST is an effective and simple tool that has been validated to be used in adult acute hospital patients (Ferguson et al. 1999). According to MST 47.9% were detected to be at risk of malnutrition. MST could be applied to the entire population due the presence of the ‘unsure’ category of the amount of lost weight (Ferguson et al. 1999).

The prevalence of malnutrition (medium + high risk) was 40% in our population according to MUST which is compatible with the prevalence of malnutrition ranging from 19-60% in hospital inpatients (Stratton et al. 2004). Some of the criteria used in MUST were not applicable to the population studied such as “acute disease status causing no or likely to cause no nutritional intake for more than 5 days”, giving a low prevalence of malnutrition than actual. Since majority of South Asians were unable to recall their usual weight or lost weight (Shirodkar and Mohandas 2005) MUST could not be utilized for the entire population to assess the nutritional status. Although we reported NRS values for the whole population, weight loss values were limited and most of scores were derived from food intake data. Among free living adults, less than quarter had known their body weight accurately in Sri Lanka (Jayawardena et al. 2014). A study done on general surgery patients has identified 6.1% prevalence of malnutrition according to NRS (Gur et al. 2009). In a another study 31.5% were identified as at high risk of malnutrition and malnutrition was more prevalent in general medical wards than in surgical patients (38.6% vs.19.1%; P < 0.001) (Giryes et al. 2012). The patients who were unable to comply with measuring height and weight directly were excluded from this study, thus excluding high risk subgroup of patients (Giryes et al. 2012). SNAQ is considered as a reliable and a cost effective malnutrition screening tool suitable to be used in both hospital inpatients and outpatients (Kruizenga et al. 2005b; Neelemaat et al. 2008). Kruizenga et al. has also demonstrated that malnutrition is associated with poor health status and care complexity. Twenty nine percent patients who had SNAQ score of at least 3 indicating severe malnutrition risk on admission, had poorer quality of life, poorer physical functioning, a lower fat free mass index, and higher care complexity. Thus malnutrition is an imperative indicator of general health status of the patients (Kruizenga et al. 2006). In this study, we found 17.7% patients to be severely malnourished and 5% to be moderately malnourished requiring nutritional intervention. Since some of the parameters assessed in MNA, MST and SGA tools were not applicable to the entire population, selection of a tool which can use for the entire population or development of a population specific malnutrition screening tool would be appropriate.

In summary, although malnutrition among cardiac patients is highly prevalent, the present study demonstrates that there is a wide variation in the nutritional status when assessed by widely used internationally recognized tools. Even though some tools are proved to be reliable to detect malnutrition accurately for various populations, each tool has to be validated before embarking to assess malnutrition in cardiac patients.

## Electronic supplementary material

Additional file 1:: **Distribution of the patients within each nutrition screening and assessment tool (online supplementary).** (DOC 144 KB)
