# Supplementary material for: Prevalence of hospital malnutrition among cardiac patients: results from six nutrition screening tools
Source: Springerplus. 2014 Aug 7;3:412. doi: 10.1186/2193-1801-3-412 (PMC4138316; doi:10.1186/2193-1801-3-412)
Supplement: Supplementary file 1 — Additional file 1:: Distribution of the patients within each nutrition screening and assessment tool (online supplementary). (DOC 144 KB) [file 40064_2014_1129_MOESM1_ESM.doc]

| **Additional file 1: Distribution of the patients within each nutrition screening and assessment tool (online supplementary)** | | | | | |
| --- | --- | --- | --- | --- | --- |
| Nutrition screening tool | | Parameter assessed | | Number | % |
| MUST (n=113, 21.5%) | | BMI (kg/m^2^) | >20 | 99 | 87.6 |
|  |  |  | 18.5-20 | 7 | 6.2 |
|  |  |  | <18.5 | 7 | 6.2 |
|  |  | Unplanned weight loss in past 3-6 months (%) | <5 | 75 | 66.4 |
|  |  |  | 5-10 | 21 | 18.6 |
|  |  |  | >10 | 17 | 15.0 |
|  |  | Patients is acutely ill and there has been or is likely to be no nutritional intake for>5 days | Yes | 0 | 0.0 |
|  |  |  | No | 113 | 100.0 |
| NRS (n=526, 100.0%) | | Normal | Food intake at a normal requirement | 230 | 43.7 |
|  | | Mild | Weight loss >5% in 3 months | 12 | 4.05 |
|  |  |  | Food intake 50-75% of normal requirement in preceding week | 42 | 14.18 |
|  |  | Moderate | Weight loss >5% in 2 months | 6 | 2.02 |
|  |  |  | BMI 18.5-20.5 + impaired general condition | 72 | 24.32 |
|  |  |  | Food intake 25-50% of normal requirement in preceding week | 102 | 34.46 |
|  |  | Severe | Weight loss >5% in 1 months | 8 | 2.70 |
|  |  |  | Weight loss >15% in 3 months | 2 | 0.68 |
|  |  |  | BMI <18.5 + impaired general condition | 50 | 16.89 |
|  |  |  | Food intake 0-25% of normal requirement in preceding week | 90 | 30.41 |
| MNA (n=526, 100.0%) | | Food intake declined over the past 3 months due to loss of appetite, digestive problems, chewing, or swallowing difficulties | Severe loss of appetite | 4 | 0.8 |
|  |  |  | Moderate loss of appetite | 204 | 38.8 |
|  |  |  | No loss of appetite | 318 | 60.5 |
|  |  | Weight loss during last 3 months | Weight loss greater than 3 kg | 13 | 2.5 |
|  |  |  | Does not know | 471 | 89.5 |
|  |  |  | Weight loss between 1-3 kg | 15 | 2.9 |
|  |  |  | No weight loss | 27 | 5.1 |
|  |  | Mobility | Bed or chair bound | 0 | 0.0 |
|  |  |  | Able to get out of bed/chair, but does not go out | 120 | 22.8 |
|  |  |  | Goes out | 406 | 77.2 |
|  |  | Has suffered psychological stress or acute disease | Yes | 1 | 0.2 |
|  |  |  | No | 525 | 99.8 |
|  |  | Neuropsychological problems | Severe dementia or depression | 0 | 0.0 |
|  |  |  | Mild dementia | 0 | 0.0 |
|  |  |  | No psychological problems | 526 | 100.0 |
|  |  | BMI (kg/m^2^) | BMI <19 | 61 | 11.6 |
|  |  |  | BMI 19-21 | 89 | 16.9 |
|  |  |  | BMI 21-23 | 85 | 16.2 |
|  |  |  | BMI >23 | 291 | 55.3 |
| SNAQ (n=119, 22.6%) | | Unintentional weight loss | > 6 kg in the past 6 months | 18 | 15.1 |
|  |  |  | > 3 kg in the past 1 month | 9 | 7.6 |
|  |  |  | No weight loss | 92 | 77.3 |
|  |  | Loss of appetite | Yes | 43 | 36.1 |
|  |  |  | No | 76 | 63.9 |
|  |  | Use of supplemental drinks or tube feeding over last month | Yes | 0 | 0.0 |
|  |  |  | No | 119 | 100.0 |
| MST (n=526, 26.4%) | | Unintentional weight loss | No | 271 | 51.5 |
|  |  |  | Unsure | 10 | 1.9 |
|  |  | If yes, lost weight | 1-5 | 8 | 1.5 |
|  |  |  | 6-10 | 9 | 1.7 |
|  |  |  | 11-15 | 1 | 0.2 |
|  |  |  | >15 | 0 | 0.0 |
|  |  |  | Unsure | 227 | 43.3 |
|  |  | Poor dietary intake because of loss of appetite | No | 332 | 63.1 |
|  |  |  | Yes | 194 | 36.9 |
| SGA (n=526, 100.0%) | History | Unintentional weight loss | Yes | 245 | 46.6 |
|  |  |  | Unsure | 10 | 1.9 |
|  |  |  | No | 271 | 51.5 |
|  |  | Poor dietary intake | Yes | 374 | 71.1 |
|  |  |  | No | 152 | 28.9 |
|  |  | Low calorie diet | Yes | 371 | 70.5 |
|  |  |  | No | 155 | 29.5 |
|  |  | Nonsolid low calorie diet | Yes | 86 | 16.3 |
|  |  |  | No | 440 | 83.7 |
|  |  | Liquid diet >15 days or IV solution >5 days | Yes | 2 | 0.4 |
|  |  |  | No | 524 | 99.6 |
|  |  | Fasting >5 days | Yes | 0 | 0.0 |
|  |  |  | No | 526 | 100.0 |
|  |  | Persistent change >30 days | Yes | 92 | 17.5 |
|  |  |  | No | 434 | 82.5 |
|  |  | Dysphagia and / or odynophagia | Yes | 42 | 8.0 |
|  |  |  | No | 484 | 92.0 |
|  |  | Nausea | Yes | 96 | 18.3 |
|  |  |  | No | 430 | 81.7 |
|  |  | Vomiting | Yes | 45 | 8.6 |
|  |  |  | No | 481 | 91.4 |
|  |  | Diarrhea | Yes | 28 | 5.3 |
|  |  |  | No | 498 | 94.7 |
|  |  | Anorexia, bloating, abdominal pain | Severe | 4 | 0.8 |
|  |  |  | Moderate | 204 | 38.8 |
|  |  |  | Normal | 318 | 60.5 |
|  |  | Functional capacity | Bed or chair bound | 120 | 22.8 |
|  |  |  | Less than normal | 406 | 77.2 |
|  |  | Diagnosis related stress level | High | 65 | 12.4 |
|  |  |  | Moderate | 177 | 33.5 |
|  |  |  | Low | 284 | 54.0 |
|  | Examination | Loss of subcutaneous fat | Severe | 2 | 0.4 |
|  |  |  | Moderate | 55 | 10.5 |
|  |  |  | Normal | 469 | 89.2 |
|  |  | Loss of striated muscle | Severe | 2 | 0.4 |
|  |  |  | Moderate | 46 | 8.7 |
|  |  |  | Normal | 478 | 90.9 |
|  |  | Sacral oedema | Severe | 2 | 0.4 |
|  |  |  | Moderate | 83 | 15.8 |
|  |  |  | Normal | 441 | 83.8 |
|  |  | Ascitis | Severe | 14 | 2.7 |
|  |  |  | Moderate | 102 | 19.4 |
|  |  |  | Normal | 410 | 77.9 |
|  |  | Ankle oedema | Severe | 14 | 2.7 |
|  |  |  | Moderate | 102 | 19.4 |
|  |  |  | Normal | 410 | 77.9 |
